# Comparative Genomics and Characterization of Hybrid Shigatoxigenic and Enterotoxigenic *Escherichia coli* (STEC/ETEC) Strains

**DOI:** 10.1371/journal.pone.0135936

**Published:** 2015-08-27

**Authors:** Outi Nyholm, Jani Halkilahti, Gudrun Wiklund, Uche Okeke, Lars Paulin, Petri Auvinen, Kaisa Haukka, Anja Siitonen

**Affiliations:** 1 Bacterial Infections Unit, Department of Infectious Diseases, National Institute for Health and Welfare (THL), Helsinki, Finland; 2 Department of Microbiology and Immunology, University of Gothenburg, Gothenburg, Sweden; 3 Institute of Biotechnology, University of Helsinki, Helsinki, Finland; 4 Department of Food and Environmental Sciences, University of Helsinki, Helsinki, Finland; University of Osnabrueck, GERMANY

## Abstract

**Background:**

Shigatoxigenic *Escherichia coli* (STEC) and enterotoxigenic *E*. *coli* (ETEC) cause serious foodborne infections in humans. These two pathogroups are defined based on the pathogroup-associated virulence genes: *stx* encoding Shiga toxin (Stx) for STEC and *elt* encoding heat-labile and/or *est* encoding heat-stable enterotoxin (ST) for ETEC. The study investigated the genomics of STEC/ETEC hybrid strains to determine their phylogenetic position among *E*. *coli* and to define the virulence genes they harbor.

**Methods:**

The whole genomes of three STEC/ETEC strains possessing both *stx* and *est* genes were sequenced using PacBio RS sequencer. Two of the strains were isolated from the patients, one with hemolytic uremic syndrome, and one with diarrhea. The third strain was of bovine origin. Core genome analysis of the shared chromosomal genes and comparison with *E*. *coli* and *Shigella* spp. reference genomes was performed to determine the phylogenetic position of the STEC/ETEC strains. In addition, a set of virulence genes and ETEC colonization factors were extracted from the genomes. The production of Stx and ST were studied.

**Results:**

The human STEC/ETEC strains clustered with strains representing ETEC, STEC, enteroaggregative *E*. *coli*, and commensal and laboratory-adapted *E*. *coli*. However, the bovine STEC/ETEC strain formed a remote cluster with two STECs of bovine origin. All three STEC/ETEC strains harbored several other virulence genes, apart from *stx* and *est*, and lacked ETEC colonization factors. Two STEC/ETEC strains produced both toxins and one strain Stx only.

**Conclusions:**

This study shows that pathogroup-associated virulence genes of different *E*. *coli* can co-exist in strains originating from different phylogenetic lineages. The possibility of virulence genes to be associated with several *E*. *coli* pathogroups should be taken into account in strain typing and in epidemiological surveillance. Development of novel hybrid *E*. *coli* strains may cause a new public health risk, which challenges the traditional diagnostics of *E*. *coli* infections.

## Introduction

Shigatoxigenic *Escherichia coli* (STEC) and other diarrheagenic *E*. *coli* (DEC) cause diarrheal disease in humans [1]. STEC cause bloody or non-bloody diarrhea. The infection may result in severe sequelae, such as hemolytic uremic syndrome (HUS). STEC produce one or two types of Shiga toxin (Stx1 and Stx2 encoded by the genes *stx*_*1*_ and *stx*_*2*_), which are responsible for the toxic effects in the host.

Several other DEC pathogroups have been established based on the pathogroup-associated virulence traits [1]. Enterotoxigenic *E*. *coli* (ETEC) cause watery diarrhea by producing heat-labile LT (encoded by *elt*) and/or heat-stable ST (encoded by *estIa* porcine variant and/or *estIb* human variant) enterotoxin. Enteropathogenic *E*. *coli* (EPEC) produces characteristic histopathology known as attaching and effacing on intestinal cells. Enteroinvasive *E*. *coli* (EIEC) is associated with invasive, bloody diarrhea resembling that caused by *Shigella* spp. Enteroaggregative *E*. *coli* (EAEC) harbors the mechanism for aggregative-adherence pattern mediated by aggregative adhesive fimbriae. EAEC is increasingly recognized as a diarrheal pathogen in developing countries.

STEC and other DECs are able to acquire virulence genes via horizontal gene transfer from other pathogroups leading to the development of *intermediate* or *hybrid* pathogroups [2–3]. A hybrid of EAEC/STEC O104:H4 caused a large outbreak with severe disease and deaths in Germany in 2011 [4]. Hybrids of STEC/ETEC have recently been reported in Germany, United States, and Slovakia [5–7], some of which have been associated with human disease [7]. In our previous studies, we have identified STEC/ETEC hybrid strains from patients and animals in Finland [8] and from animal derived food in Burkina Faso [9].

*E*. *coli* is a genetically versatile species. Strains within a single pathogroup can originate from different genetical backgrounds [10–13]. Among STEC, the Locus of Enterocyte Effacement (LEE) negative strains have evolved and acquired *stx*-phages multiple times [14]. In addition, *E*. *coli* strains belonging to different phylogenetic lineages can independently evolve into enterohemorrhagic form of STEC by acquiring phages and other integrative elements, such as LEE, essential for the virulence properties [11]. Also the ETEC pathogroup consists of strains of polyphyletic origin [15]. Multi locus sequence typing (MLST) has revealed that ETEC strains originate from different evolutionary lineages indicating that the acquisition of the *elt* or *est* genes may be enough to make an ETEC strain [15]. In addition, the prototypical ETEC strain H10407 chromosome is almost identical with the chromosome of *E*. *coli* K-12 strain MG1655 suggesting that the main event in the emergence of ETEC from *E*. *coli* is the acquisition of virulence plasmids carrying *elt* or *est* [16]. The variability in virulence gene and colonization factor combinations highlights the genomic diversity within the ETEC pathogroup [12]. These findings suggest that ETEC consists of genetically heterogeneous group of strains that have gained the ETEC-associated virulence genes by horizontal gene transfer. However, recent evidence, based on the sequence analysis of 362 ETEC isolates, shows that persistent plasmid-chromosomal background combinations exist in certain phylogenetic lineages [17].

Genomics and phylogeny of hybrid *E*. *coli* strains have not been studied widely. An exception is the German outbreak strain EAEC/STEC O104:H4, which was shown to form a distinct clade with other O104:H4 strains among EAEC and *E*. *coli* indicating that the outbreak strain has the chromosomal backbone similar to EAEC O104:H4 group [18]. In a recent study, STEC/ETEC hybrid strains of several serotypes were not found phylogenetically related [14]. This suggests that these strains may have arisen from several genetic backgrounds.

In the present study, we investigated human and bovine STEC/ETEC hybrid strains to determine their phylogenetic position among *E*. *coli* and to define the similarities and differences in their gene contents and virulence properties related to other DEC pathogroups. We used whole genome sequencing and whole genome mapping for comparative genomics between the STEC/ETEC genomes and the reference genomes of pathogenic and commensal *E*. *coli* and *Shigella* spp. It is crucial to understand the phylogeny of pathogenic bacteria to evaluate how they have evolved and to monitor the emergence of potential new pathogens.

## Materials and Methods

### Bacterial strains and reference genomes

The whole genomes of three *E*. *coli* strains possessing both STEC- and ETEC-associated virulence genes *stx*_*1*_ or *stx*_*2*_ and *estIa* were sequenced. The strains have been described in our previous studies [8–9]. The strain IH53473 (serotype O101:H^-^) was isolated from a 1.9-year-old infant with HUS in Finland and the strain IH57218 (serotype O2:H27) was isolated from a 7.3-year-old child with diarrhea in Finland [8] (S1 Table). The strain FE95160 (serotype O2:H2) was isolated from a bovine intestine sample in Burkina Faso [9] (S1 Table). For comparative genomics, publicly available complete and draft genomes of different DEC pathogroups, extraintestinal pathogroups, commensal strains, laboratory-adapted strains, and *Shigella* spp. strains were downloaded from GenBank (http://www.ncbi.nlm.nih.gov/genbank) (S1 Table).

### DNA extraction and whole genome sequencing

Genomic DNA was extracted using QIAGEN Genomic Tip 100/G (QIAGEN, Gaithersburg, MD, USA) according to the manufacturer’s instructions. After the extraction, the intactness of the genomic DNA was verified by agarose gel electrophoresis and the quantity of the genomic DNA was measured by Qubit 2.0 Fluorometer (Invitrogen, Carlsbad, CA, USA).

Sequencing libraries were constructed according to the manufacturer’s (Pacific Biosciences, Menlo Park, CA, USA) protocol. Sequencing was done on the PacBio RS instrument (Pacific Biosciences) with P4/C2 chemistry.

### *De novo* genome assembly, genome annotation, and validation of the assembly by whole genome mapping

The data collected from the PacBio RS instrument were processed and filtered using the single molecule real-time (SMRT) analysis software suite (Pacific Biosciences). Data were filtered by read quality (> 0.75) and read length (> 1000 bp). When processing continuous long read (CLR) data, raw reads from the SMRT Cells were split on adapter sequence resulting in ≥ 1 subread or CLR per zero-mode waveguides (ZMW). For SMRT *de novo* assembly, the HGAP pre-assembly workflow was used to generate long and highly accurate sequences. This was accomplished by mapping single pass reads to seed reads, which represent the longest portion of the read length distribution. Subsequently, a consensus sequence of the mapped reads was generated resulting in long and highly accurate fragments of the target genome. We further pruned reads from this pre-assembly pipeline that were < 3500bp. The pre-assembled reads were output to FASTQ format and further error corrected using the PacBioToCA utility in Celera Assembler (CA 7.0). The reads were then assembled with the CA 7.0 assembler using the BOGART algorithm with mer size of 25. Scaffolding was carried out using the CGW algorithm of the CA 7.0 assembler.

The final number of contigs per genome after the assembly was 10 for IH53473, 17 for IH57218, and 43 for FE95160. The three draft genomes were uploaded into Galaxy/CRS4 (Orione) [19] for automatic annotation with PROKKA (version 1.4.0) [20] using default settings. The whole genome sequence reads were deposited at NCBI SRA (study accession no. PRJNA269579). The draft genomes were deposited at NCBI Whole Genome Shotgun (WGS) database under accession number LFZH00000000 for IH53473, LFZJ00000000 for IH57218, and LFZI00000000 for FE95160.

Whole genome mapping (i.e. optical mapping) was used to correct the order of contigs and to detect any misassemblies. For this, the chromosomal DNA was digested using *Nco*I restriction enzyme. Whole genome maps were produced using Argus Optical Mapping System (OpGen Inc., Gaithersburg, MD, USA) as previously described [21]. To compare the sequence contigs to the respective whole genome map, the sequence contigs were restricted with *Nco*I *in silico* and the contigs were aligned against the map using MapSolver 3.2.0 software (OpGen Inc.).

### Extraction of virulence genes and ETEC colonization factors

The genomes were screened for a broad spectrum of known and characterized *E*. *coli* virulence genes (S2 Table) using Ridom SeqSphere+ program (Ridom GmbH, Münster, Germany). The target loci were imported from annotated publicly available sources (e.g. genomes, plasmids, coding sequences). Required thresholds for gene identification were ≥80% identity to reference sequence and ≥99% alignment with reference sequence. If a sequencing error was suspected, the target sequence was verified by Sanger sequencing. ETEC colonization factors and *stx*_*1*_ and *stx*_*2*_ subtypes were identified by *in silico* primer search using Geneious 6.0.5 software (Biomatters Ltd, Auckland, New Zealand). Primer sequences for ETEC colonization factors CFA/I, CS1, CS2, CS3, CS4, CS5, CS6, CS7, CS8, CS12, CS13, CS14, CS15, CS17, CS17-19, CS20, CS21, and CS22 were obtained from previously published articles [22–24]. Primer sequences for three *stx*_*1*_ and seven *stx*_*2*_ subtypes were obtained from the published PCR protocol [25]. *stx*_*1*_ and *stx*_*2*_ subtyping was performed to compare *in silico* typing to the previous *stx*_*1*_ and *stx*_*2*_ subtyping PCR results [8–9].

### *In silico* MLST, phylogrouping, and serotyping

The allelic profiles of the seven genes, *adk*, *fumC*, *gyrB*, *icd*, *mdh*, *purA*, *recA*, used in the published protocol for *E*. *coli* MLST [26], were extracted using *E*. *coli* MLST application in Ridom SeqSphere+ program (Ridom GmbH) to determine MLST sequence types (ST) of the STEC/ETEC genomes and reference genomes.

Phylogroups of the STEC/ETEC genomes and reference genomes except *Shigella* spp. were determined *in silico* using phylotyping primers for the *E*. *coli* phylogroups A, B1, B2, C, D, E, F, and cryptic clades I-V [27–28] and Geneious 6.0.5 software (Biomatters Ltd).

The three STEC/ETEC strains were previously serotyped using the traditional O- and H-antigen agglutination [8–9]. We now compared the previous results with the *in silico* serotyping results. The genes responsible for the expression of O- and H-antigens were detected from the genomes. The primers and probes to detect the respective O- and H-sequences were previously published [29–30]. The primer and probe search were done using Geneious 6.0.5 software (Biomatters Ltd).

### Identification of prophage regions and *stx*-phage integration sites and testing for the production of Stx and ST toxins

PHAST tool (phast.wishartlab.com) [31] was used to identify prophage sequences in the STEC/ETEC genomes. A prophage region was considered to be intact if the completeness score was above 90, questionable if the score was between 60 and 90, and incomplete if the score was less than 60. The integration sites for the *stx*-phages were determined manually. The *stx* genes were located in the assembled contigs. Starting from the *stx* gene, the sequence upstream and downstream was screened for the phage-related genes using the BLAST tool [32] and Geneious 6.0.5 software (Biomatters Ltd). If the phage sequence was not contiguous, the phage was reconstructed joining the sequence contigs together with the guidance of the whole genome map. The interrupted gene adjacent to the phage integrase was designated as the phage integration site.

Production and titers of Stx and ST were determined. Stx was tested on the Vero cell assay at Statens Serum Institut (Copenhagen, Denmark). ST was determined using the GM1-ELISA method as previously described [33–34]. The prototypical ETEC strain H10407 was used as a control in the ST titration.

### Identification of plasmid-associated sequences

PlasmidFinder 1.2 [35] was used to identify the presence of plasmids in the three STEC/ETEC genomes. Identification was based on the detection of replicon sequences belonging to several known plasmid incompatibility (Inc) groups. The threshold for identification was set to 80%. The locations of the plasmid Inc groups were compared to the locations of known plasmid-associated genes *estIa*, *hlyA*, *espP*, and *astA* in the contigs of the draft genomes. PROKKA annotation reports were also utilized in the survey of the possible plasmid sequences.

### Comparative genomics

To generate a phylogenetic tree depicting positions of the three STEC/ETEC strains, the genomes were compared with 73 published *E*. *coli* and *Shigella* spp. reference genomes, both completed and draft genomes (S1 Table). Phylogenetic analysis based on the genes common to all the genomes included in the comparison was performed using Ridom SeqSphere+ program (Ridom GmbH). Gene nomenclature used in the analysis was based upon the strain ETEC H10407 (accession no. FN649414.1). All the annotated coding sequences were imported from the ETEC H10407 genome to create a task template for core genome MLST (cgMLST). Required thresholds for gene identification were ≥90% identity to reference sequence and ≥99% alignment with reference sequence. If a single target gene resulted in more than one match in the genome, the whole target was excluded from the analysis. Altogether, 1341 targets were determined as the shared genes. These 1341 targets of all the 76 genomes included in the cgMLST analysis were concatenated into 76 continuous sequences and exported from Ridom SeqSphere+ as a multi-fasta file. The sequences were uploaded into Galaxy/CRS4 (Orione) [19] and aligned with MAFFT (version 0.1) [36]. The alignment was imported into Geneious 6.0.5 software. UPGMA dendrogram including BootStrap confidence values was produced using Jukes-Cantor genetic distance model within the Geneious tree builder tool. Among the reference genomes, there were five draft genomes that were previously characterized as STEC/ETEC hybrids [14,37] and 14 draft genomes that represented 14 phylogenetic lineages L1-L14 of the ETEC pathogroup [17].

Restriction enzyme *Nco*I based whole genome maps were used to determine the degree of genomic identity between the three STEC/ETEC chromosomes. The maps were compared with each other and similarity percentage was calculated for each pair using MapSolver 3.2.0 software (OpGen Inc.). Whole genome maps were also used to generate phylogenetic tree where the three STEC/ETEC whole genome maps were compared with *in silico Nco*I restricted maps generated from completed reference chromosomal sequences (S1 Table) using UPGMA algorithm.

## Results

### *De novo* genome assemblies

All three STEC/ETEC genomes remained as draft genomes including several gaps when aligned to whole genome maps produced from the chromosomal DNA. The chromosome sizes derived from whole genome maps were as follows: 5 097 783 bp, 5 123 796 bp, and 4 907 103 bp for strains IH53473, IH57218, and FE95160, respectively. The sequence contigs contained the plasmid DNA but the maps were only of the chromosomal DNA. In the genome IH57218, a misassembled area inside one contig was detected when aligned against the whole genome map. The other two genomes were assembled correctly according to the alignments against the maps.

### Virulence genes harbored by STEC/ETEC strains

All three STEC/ETEC strains harbored multiple virulence genes (Table 1). All the strains carried the genes *clyA* encoding cytolysin and *shiA* encoding shikimate transporter. The human strain IH53473 was the only strain possessing the gene *espP*, which belongs to the group of Serine Protease Autotransporters of *Enterobactericeae* (SPATE). No other SPATEs were detected. IH53473 possessed also genes *irp1*, *irp2*, and *fyuA* encoding yersiniabactin biosynthetic proteins and a receptor. IH53473 was positive for LEE pathogenicity island, which contains the gene *eae* encoding intimin, *espA* and *espD* encoding translocators, and *escV*, *espF*, and *espH* encoding type III secretion system structure and effector proteins. The human strain IH57218 was positive for *Shigella* enterotoxin 2. The bovine strain FE95160 was positive for *astA* encoding EAEC heat-stable enterotoxin I and *aai* pathogenicity island encoding type VI secretion system.

**Table 1:** Virulence genes in the STEC/ETEC genomes.

Gene/Target	Product/Function	IH53473	IH57218	FE95160
stx1A	Shiga toxin 1 subunit A	-	-	+
stx1B	Shiga toxin 1 subunit b	-	-	+
stx2A	Shiga toxin 2 subunit A	+	+	-
stx2B	Shiga toxin 2 subunit b	+	+	-
sta1 (estIa)	Heat-stable enterotoxin sti-a/st-p precursor	frame shift	+	+
astA	EAEC heat-stable enterotoxin I	-	-	+
hlyA (ehxA)	Hemolysin A	+	+	+
hlyB (ehxB)	Hemolysin B	+	+	+
hlyC (ehxC)	Hemolysin C	+	+	+
hlyD (ehxD)	Hemolysin D	+	+	+
eae	Intimin	+	-	-
escV	T3SS structure protein EscV	+	-	-
espA	Translocator EspA	+	-	-
espD	Translocator EspD	+	-	-
espF	T3SS effector EspF	+	-	-
espH	T3SS effector EspH	+	-	-
espP	Extracellular serine protease, autotransporter, SPATE	+	-	-
eaeH	Putative adhesin	+	+	+
yfaL	Putative outer membrane autotransporter adhesin	+	+	-
clyA	Cytolysin A	+	+	+
ShET-2	Shigella enterotoxin ShET-2 domain containing protein	-	+	-
ter	Tellurite resistance	+	+	+
ecpA	Common pilus subunit	+	+	+
fimH	Type 1 fimbria	-	+	+
shiA	Shikimate transporter	+	+	+
irp1	Yersiniabactin biosynthetic protein	+	-	-
irp2	Yersiniabactin biosynthetic protein	+	-	-
fyuA	Pesticin, yersiniabactin receptor protein	+	-	-
aai pathogenicity island	Type VI secretion system	-	-	+

All the strains were negative for ETEC colonization factors. However, the strains harbored some of the tested virulence genes contributing to adherence: putative adhesin gene *eaeH*, putative outer membrane autotransporter adhesin gene *yfaL*, type 1 fimbria gene *fimH*, and common pilus subunit gene *ecpA* (Table 1).

The results of *in silico stx*_*1*_ and *stx*_*2*_ subtyping were consistent with the previous results obtained by PCR: IH53473 *stx*_*2a*_, IH57218 *stx*_*2a*_, FE95160 *stx*_*1a*_.

The virulence gene analysis revealed that strain IH53473 possessed a frame shift mutated *estIa* gene while the other two STEC/ETEC hybrids had intact *estIa* genes. To verify whether the mutation was real or a sequencing error, the gene *estIa* was amplified by PCR from all the strains and the PCR products were sequenced by Sanger sequencing. The result was confirmed: *estIa* in IH53473 had a single nucleotide deletion which resulted in a frame shift mutation and produced a premature stop codon. The translated polypeptide would be only 53 amino acids long, whereas the full length polypeptide consists of 73 amino acids.

### *In silico* MLSTs, phylogroups, and serotypes

The STs of the human strains were of the previously established types: ST330 for IH53473 and ST10 for IH57218. The ST of the bovine strain FE95160 was novel. It was submitted to the *E*. *coli* MLST database (http://mlst.warwick.ac.uk/mlst/dbs/Ecoli) and was assigned with a new ST number ST4123.

Both human strains belonged to the *E*. *coli* phylogroup A. The bovine strain belonged to the cryptic clade I.

O-grouping results were consistent with the previous results: IH53473 O101, IH57218 O2, and FE95160 O2. However, H-typing results were different in two strains. IH53473, which had previously been typed as H^-^/non-motile had primer and probe binding sites for H33. FE95160, which had previously been typed as H2 had primer and probe binding sites for H25. H-typing result of strain IH57218 was consistent with the previous result H27.

### Identified prophage regions and *stx*-phage integration sites and production of Stx and ST toxins

For strain IH53473, 16 prophage regions were identified, of which nine regions were intact, three regions were incomplete, and four regions were questionable. For strain IH57218, 17 prophage regions were identified, of which 12 regions were intact, and five regions were incomplete. For strain FE95169, 10 prophage regions were identified, of which seven regions were intact, and three regions were incomplete.

The integration sites for the *stx*_*2a*_-phages in IH53473 and IH5728 were identified in *wrbA* locus. Both phages were similar to phage P13374 (accession no. HE664024) by BLAST search. The integration site for the *stx*_*1a*_-phage in strain FE95160 was between the genes *ybhC* and *ybhB*. The phage remained unidentified as no hits were found by BLAST search.

All the three STEC/ETEC strains expressed Stx with titers of 1:100,000 dilution. IH57218 and FE95160 produced STIa. The STIa titers were 168 ng/ml for IH57218, 40 ng/ml for FE95160, and 253 ng/ml for ETEC H10407 control strain. IH53473 did not produce STIa.

### Plasmid-associated sequences

PlasmidFinder indicated several plasmid replicon sequences of known Inc groups in the STEC/ETEC genomes. IH53473 had three plasmid replicons: IncQ2, IncFII(29), and IncXI. The plasmid-associated genes *estIa*, *hlyA*, and *espP* were placed in the same contig as IncFII(29). IH57218 had one plasmid replicon: IncFII. The plasmid-associated genes *estIa* and *hlyA* were placed in a different contig than IncFII. FE95160 had two plasmid replicons: IncFII(pSE11), and IncFIB(AP001918). The plasmid-associated genes *estIa*, *hlyA*, and *astA* were placed in the same contig as IncFIB(AP001918). According to the PROKKA annotation reports of IH53473 and FE95160, several plasmid-associated genes, such as RepFIB replication protein A, plasmid partitioning protein B, and plasmid stability protein, were located in the same contigs as the virulence genes *estIa*, *hlyA*, espP, and *astA*. According to the PROKKA annotation report of IH57218, several plasmid-associated genes were also found in the same contig with *estIa* and *hlyA*. However, no origin of plasmid replication was present in that contig but it was located in another one.

### Comparative genomics analyses

**Fig 1:**
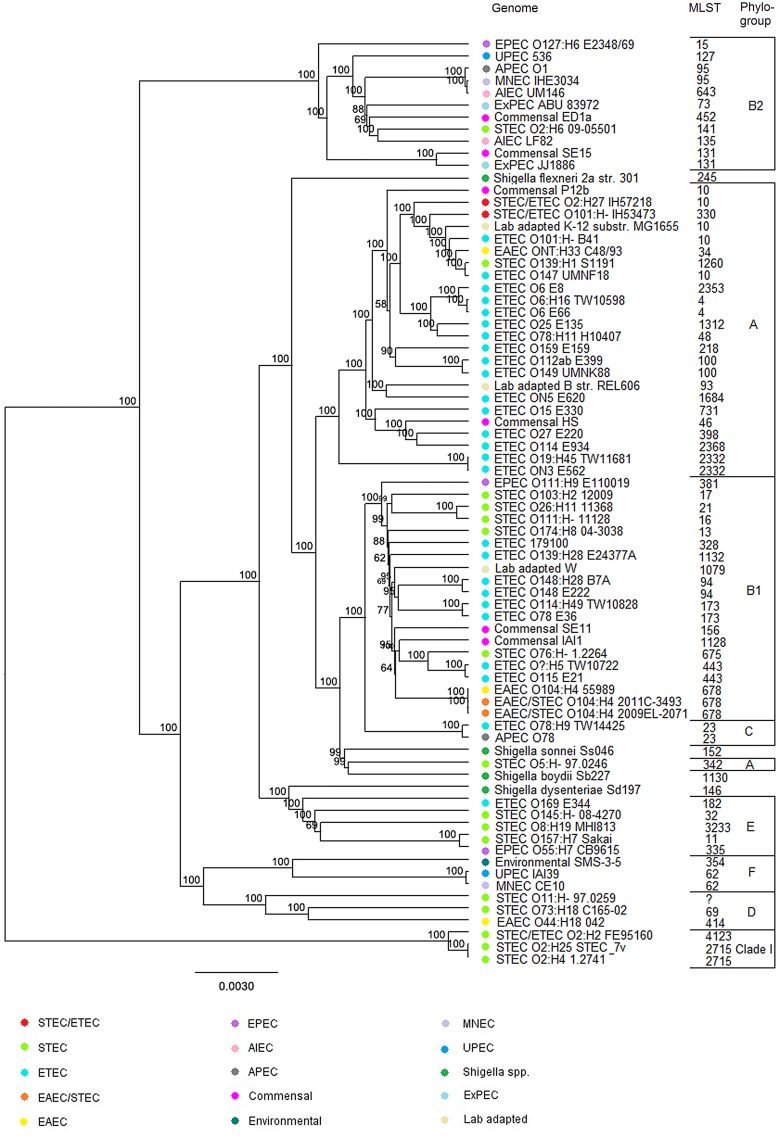
Phylogenetic placement of STEC/ETEC strains using core genome MLST and sequence alignment. UPGMA tree based on aligned sequences of the defined *E*. *coli* core genome genes (n = 1341) showing the phylogenetic relationship of the three STEC/ETEC genomes and 73 additional *E*. *coli* and *Shigella* spp. strains. The different pathogroups, STEC, ETEC, EPEC, EIEC, EAEC, AIEC (adherent/invasive *E*. *coli*), APEC (avian pathogenic *E*. *coli*), UPEC (uropathogenic *E*. *coli*), ExPEC (extraintestinal pathogenic *E*. *coli*), MNEC (meningitis causing *E*. *coli*), commensal, and environmental *E*. *coli* are marked by colors. The reference genomes STEC O139:H1 S1191, ETEC UMNF18, STEC O2:H25 7v, STEC O8:H19 MHI813, and STEC O73:H18 C165-02 were previously characterized as STEC/ETEC hybrids [14,37]. The reference genomes ETEC O6 E8, ETEC O6 E66, ETEC O78 E36, ETEC O25 E135, ETEC O115 E21, ETEC ON3 E562, ETEC O169 E344, ETEC O148 E222, ETEC O27 E220, ETEC O114 E934, ETEC O159 E159, ETEC O15 E330, ETEC O112ab E399, and ETEC ON5 E620 represent the phylogenetic lineages L1-L14 of the ETEC pathogroup, respectively [17].

The core genome phylogeny was inferred from the shared genes among a diverse set of *E*. *coli* and *Shigella* spp. genome sequences using cgMLST and sequence alignment. The analysis showed that different *E*. *coli* pathogroups are inter-mixed (Fig 1). For instance, STEC genomes can be found from nearly all branches of the UPGMA tree. The three STEC/ETEC strains did not form a single cluster. The two human STEC/ETEC strains IH53473 and IH57218 clustered with genomes representing ETEC, STEC, EAEC, and laboratory-adapted *E*. *coli*. If the cut-off point for the cluster was further extended within the genomes belonging to phylogroup A, other ETEC, laboratory-adapted and commensal *E*. *coli* genomes were included into the cluster. The bovine STEC/ETEC strain FE95160 formed a remote cluster with two STEC genomes. These three genomes belonged to cryptic clade I while other genomes included in the tree belonged to the actual phylogroups. The core genome phylogeny followed the phylogrouping results with one exception: STEC O5:H- 97.0246 genome was separated from the rest of the phylogroup A genomes.

The human STEC/ETEC strains clustered with previously characterized STEC/ETEC hybrids STEC O139:H1 S1191 and ETEC O147 UMNF18 and the bovine STEC/ETEC strain clustered with STEC O2:H25 7v. The other two previously characterized STEC/ETEC hybrids, STEC O8:H19 MHI813 and STEC O73:H18 C165-02, did not cluster with our STEC/ETEC hybrids. The ETEC reference genomes ETEC O6 E8, ETEC O6 E66, and ETEC O25 E135 representing the major ETEC lineages L1, L2, and L4, respectively, were the closest relatives of our human STEC/ETEC strains. The second closest relatives were the genomes ETEC O159 E159 and ETEC O112ab E399, which represent colonization factor negative ETEC lineages L11 and L13, respectively.

**Fig 2:**
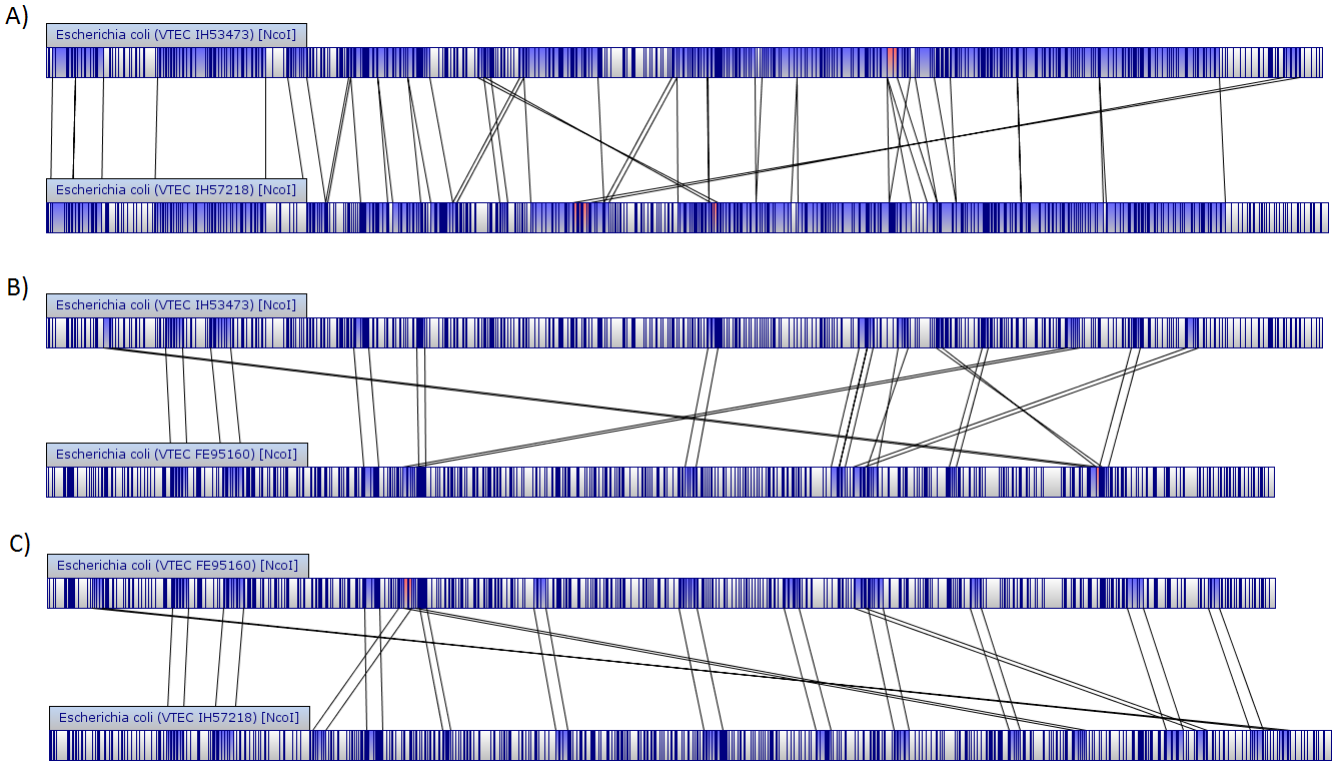
Whole genome map comparison of STEC/ETEC strains. Areas in blue are common between two maps, areas in white are unique to the map in which they are contained, and areas in red are matching more than once. (A) Comparison between IH53473 and IH57218, (B) comparison between IH53473 and FE95160, and (C) comparison between IH57218 and FE95160.

Whole genome maps of the STEC/ETEC strains were compared with each other. According to the map lengths, the human strains IH53473 and IH57218 both have approximately 5.1 Mb chromosomes. The comparison between them indicated several homological regions (Fig 2). The bovine strain FE95160 has approximately 4.9 Mb chromosome, which is notably shorter than the human STEC/ETEC chromosomes. Comparison of FE95160 with IH53473 and with IH57218 indicated only a few homologous regions between the chromosomes (Fig 2). Based on the restriction map similarity, the chromosome of IH53473 is expected to demonstrate approximately 69% identity with that of IH57218, the chromosome of IH53473 approximately 12% identity with that of FE95160, and the chromosome of IH57218 approximately 16% identity with that of FE95160 (S1 Fig).

**Fig 3:**
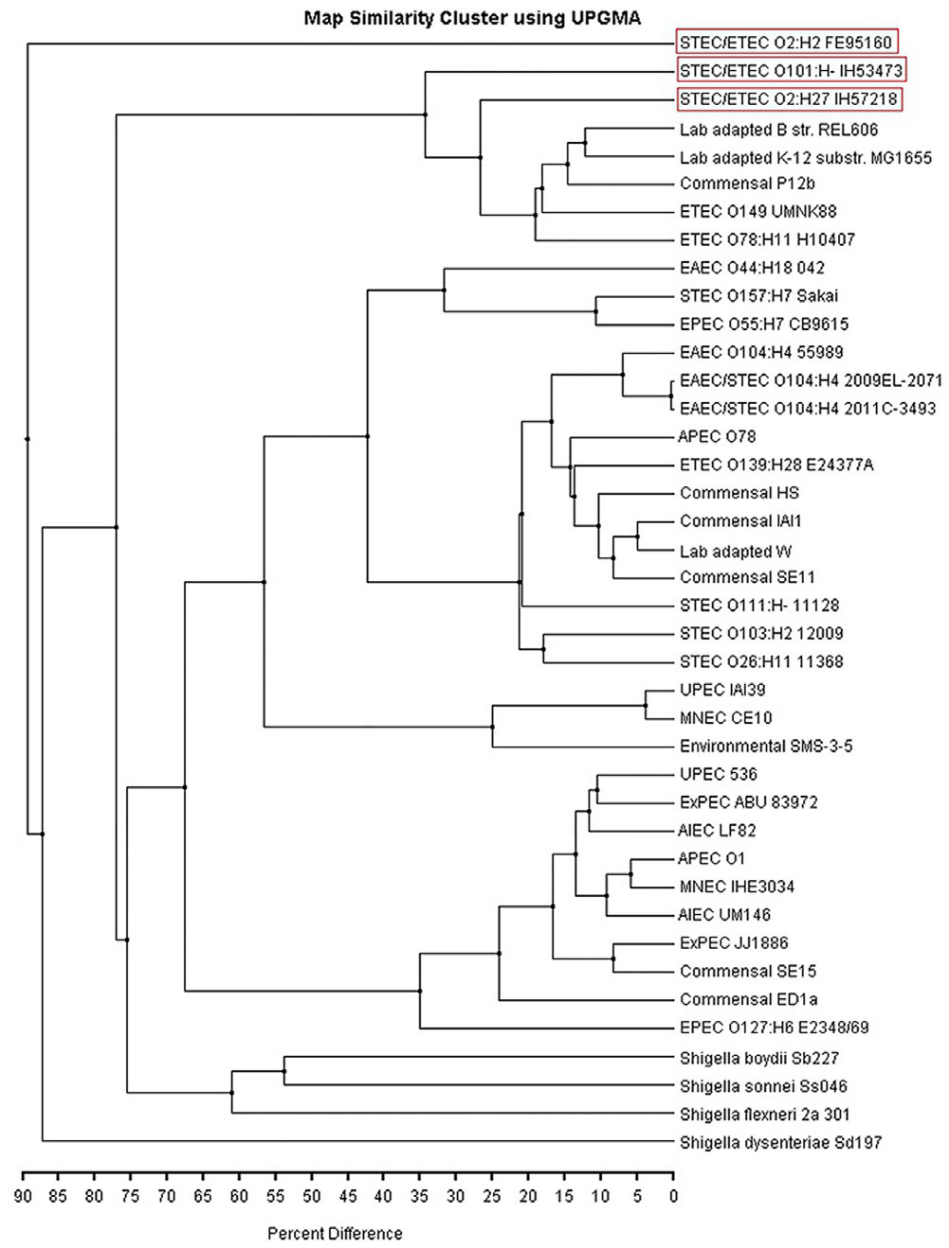
Phylogenetic placement of STEC/ETEC chromosomes using restriction-based and *in silico* whole genome maps. STEC/ETEC chromosomal maps (red boxes) were compared to other *E*. *coli* and *Shigella* spp. chromosomal maps. Similarities were calculated using UPGMA algorithm.

Whole genome maps of the STEC/ETEC chromosomes were compared to *in silico* maps of completed reference *E*. *coli* and *Shigella* spp. chromosomal sequences (Fig 3). The human strains IH53473 and IH57218 clustered with strains representing ETEC, commensal, and laboratory-adapted *E*. *coli*. However, the bovine strain FE95160 formed an outgroup in the UPGMA tree. The whole genome map similarity clustering is comparable to the clustering fashion in the cgMLST based UPGMA tree (Fig 1). Again, the STEC genomes can be seen in more than one cluster on the UPGMA tree. However, the number of genomes included into the whole genome map comparison is smaller due to the limited number of completed genomes available.

## Discussion

This study investigated the virulence gene contents of three STEC/ETEC hybrid strains and their phylogenetic position in relation to other *E*. *coli* genomes. The information obtained in this study reveals the genomic diversity of STEC/ETEC hybrid strains and contributes significantly to our understanding of genomics and virulence factor repertoire of hybrid *E*. *coli* strains.

The varying combination of multiple virulence genes harbored by the STEC/ETEC hybrids showed that the strains were a mixture of several different *E*. *coli* pathogroup-associated properties. The presence of virulence genes *stx* and *estIa* associated with two different pathogroups in our strains confirmed their hybrid status. In addition, the strains harbored other virulence genes, some of which have been associated with ETEC, STEC, and EAEC. All STEC/ETEC strains were positive for *clyA*, which encodes cytolysin A and which has been associated with ETEC pathogroup [12]. The human strain IH53473 harbored the genes *irp1*, *irp2*, and *fyuA* for two yersiniabactin biosynthetic proteins and a receptor located in a high-pathogenicity island (HPI), which is prevalent in several EAEC isolates but rarely detected in other DECs [24,38]. HPI may contribute to virulence by offering an iron scavenging system for survival in the host. One SPATE gene, *espP*, was also detected in IH53473. SPATE is a family of extracellular proteases produced by the species belonging to *Enterobacteriaceae* and they have an impact on mucosal damage and colonization [39]. The German outbreak strain EAEC/STEC O104:H4 harbored a combination of several SPATEs which may have contributed to its heightened virulence [18]. The bovine strain FE95160 was positive for *astA* encoding EAEC heat-stable enterotoxin I. Unlike the toxin name indicates, *astA* can be harbored by strains of STEC, EAEC, EPEC, ETEC, and EIEC pathogroups [40] and even extraintestinal pathogenic *E*. *coli* [41]. FE95160 was also positive for *aai* pathogenicity island encoding putative type VI secretion system, which was also detected in the German outbreak strain EAEC/STEC O104:H4 [18].

All the STEC/ETEC strains possessed some of the genes *eaeH*, *yfaL*, *ecpA*, and *fimH* associated with adhesion [12,16,42–43]. These genes may contribute to the adhesion while the strains lacked ETEC colonization factors. The genes *ecpA* and *fimH* have been associated with ETEC pathogroup [12]. It is not uncommon that ETEC strains are negative for ETEC colonization factors [24]. Thus, these colonization factor negative strains may have other virulence genes by which they can adhere. The human strain IH57218 was positive for the gene encoding *Shigella* enterotoxin 2, which can increase virulence [44]. All STEC/ETEC strains were positive for the gene *shiA* encoding shikimate transporter in a pathogenicity island involved in the suppression of host inflammatory response [45].

The phylogeny inferred from cgMLST and sequence alignment demonstrates that our STEC/ETEC hybrid strains do not form a single cluster. The phylogenetic placement of the human STEC/ETEC strains indicates a common ancestor with certain ETEC, STEC, EAEC, laboratory-adapted and commensal *E*. *coli* strains. On the contrary, the bovine strain FE95160 shares similar genetic background with two STEC genomes of bovine origin and they form a remote cluster in the phylogenetic tree. The phylogeny inferred from whole genome map similarity clustering supports these observations. Also the whole genome map comparison between the three STEC/ETEC strains is consistent with the remote phylogenetic position of the bovine strain since the human strains were shown to share more genetic elements with each other than with the bovine strain.

The previous studies have shown that both STEC and ETEC pathogroups are genetically versatile [11,14–15]. Our observations on cgMLST and whole genome map similarity clustering support this. Both STEC and ETEC genomes were found from several branches in the two UPGMA trees. It has been suggested that the acquisition of the toxin genes may be all that is required to form ETEC and there are no specific chromosomal factors prerequisite for the enterotoxigenicity [15]. However, von Mentzer and colleagues [17] recently described several robust phylogenetic lineages in the ETEC pathogroup. Lineages L1-L10 possessed certain colonization factor and toxin gene profiles whereas lineages L11-L14 were always colonization factor negative. Their data showed that toxin allele profiles and colonization factor profiles were associated with certain chromosomal background. Thus, we included 14 ETEC draft genomes representing the ETEC lineages L1-L14 from von Mentzer and colleagues’ study [17] into our cgMLST analysis to see if the STEC/ETEC genomes cluster with some of these genomes. The genomes representing the major ETEC lineages L1, L2, and L4 were the closest relatives of our human STEC/ETEC strains. The second closest relatives were the genomes representing colonization factor negative ETEC lineages L11 and L13. These results might indicate that STEC/ETEC hybrid strains also have genetic backgrounds linked with certain colonization factor and toxin gene profiles.

Even though all our STEC/ETEC strains did not cluster together, we found evidence that STEC/ETEC hybrid strains may have similarities in their chromosomal background. In our study, we combined our data with the previously sequenced five STEC/ETEC hybrid genomes in cgMLST [14,37]. Our human STEC/ETEC strains clustered with STEC O139:H1 S1191 [14], which was isolated from a pig suffering from edema disease and possesses *estIb* and *stx*_*2e*_ genes, and with ETEC O147 UMNF18 [37], which is also of porcine origin and possesses *estIa*, *estIb* and *stx*_*2e*_ genes. Our bovine STEC/ETEC clustered with STEC O2:H25 7v [14], which has been isolated from cattle feces and possesses *estIa* and *stx*_*2g*_ genes. The other two STEC/ETEC hybrids from Steyert et al. study [14], STEC O8:H19 MHI813 and STEC O73:H18 C165-02, did not cluster with the rest of the STEC/ETEC hybrids. We suggest that certain genetic background may favor the acquisition of ETEC virulence genes and *stx*-phages.

*E*. *coli* MLST ST10, which includes our human STEC/ETEC strain IH57218, is common among the ETEC strains from human origin [15]. Interestingly, there is a previous report of an UPEC strain having ST330 with the *estIa* gene [46], as is the case with our human STEC/ETEC IH53473 strain, although the latter had a frame shift mutation in the *estIa* gene. IH53473, which was shown to be SPATE *espP* positive, clustered together with an EAEC genome in cgMLST based UPGMA tree. EAEC often harbor several SPATEs, and *espP* is class I cytotoxic SPATE [39]. IH53473 was shown to possess a variety of virulence factors that may have had an effect on its pathogenic potential since the strain was isolated from a patient with HUS.

Two of the STEC/ETEC strains were able to produce both Stx and STIa. The ability to produce both toxins may result in increased virulence. All three STEC/ETEC strains had a very high Stx titer of 1:100,000 dilution. There was no difference in Stx cytotoxicity between the human and bovine STEC/ETEC strains. Human isolates possessing *stx*_*2a*_, *stx*_*2c*_, or *stx*_*2dact*_ show generally higher cell cytotoxicity compared to *stx*_*2b*_, *stx*_*2e*_, or *stx*_*2g*_ [47]. Our human STEC/ETEC strains possessing *stx*_*2a*_ were isolated from patients with HUS or diarrhea. IH53473 also carried *eae*. Clinically relevant STEC, and especially *eae* positive STEC, have shown high cytotoxicity levels compared to food isolates, which have shown more diverse cytotoxicity levels [47]. The human strain IH57218 and the control strain ETEC H10407 showed higher STIa production rate compared to the bovine strain. The human strain IH53473 produced Stx but not STIa. The result is consistent with the detected frame shift mutation in the *estIa* gene.

*In silico* O-grouping and H-typing may be used to replace agglutination-based serotyping. In the present study, O-grouping results were consistent with the previous results obtained by antiserum agglutination [8–9]. However, some of the H-grouping results were not consistent. Strain IH53473, which was previously typed as H-/non-motile had primer and probe binding sites for H33. Since the strain was not motile, the phenotypic H-antigen agglutination test could not be performed. Strain FE95160 was previously typed as H2. However, *in silico* method showed primer and probe binding sites for H25. The results may be due to the fact that the H-agglutination schema [48] has only one reaction difference between H2 and H25. *In silico* typing seems to be a good choice especially for non-motile strains which cannot be typed by agglutination due to the lack of the expression of H-antigen.

The genes *estIa* and *astA* may be plasmid- or chromosome-associated [1]. The locations of the plasmid replicon sequences identified in the STEC/ETEC genomes were associated with the locations of potentially plasmid-associated genes *estIa*, *hlyA*, *espP*, and *astA* in the genomes of IH53473 and FE95160. This may indicate that the *estIa* genes of IH53473 and FE95160 and *astA* gene of FE95160 are located on plasmids rather than the chromosome. The assembly pipeline favors the assembly of plasmid sequences in separate contigs. Also *estIa* in IH57218 genome may be plasmid-associated. Even though the plasmid replicon sequence was detected in another contig than *estIa*, this may be due to fragmentation of plasmid sequence in two separate contigs during the assembly.

All our STEC/ETEC strains possessed several prophage regions in their genomes. It is typical of STEC genomes to harbor prophages and other integrative elements [13]. The identified *stx*-phage integration sites in the STEC/ETEC genomes were the usual ones. In the IH53473 and IH57218 genomes *stx*-phage interrupted the *wrbA* gene. The gene *wrbA* encoding tryptophan repressor binging protein is a common integration site for the *stx*_*2*_ phages [49]. The FE95160 genome had the *stx*-phage integration site between the genes *ybhC* (predicted pectinesterase) and *ybhB* (predicted kinase inhibitor). We screened the *E*. *coli* reference genomes for similar cases and noticed that also *E*. *coli* UMN026 (accession no. NC_011751.1) and *E*. *coli* EDL933 (accession no. NC_002655.2) possess phages on this site.

The Stx-phages and their ability to transfer genes horizontally play an important role in the evolution of *E*. *coli* and development of STEC variants [50]. Also the plasmids carrying ST and LT toxin genes can be transferred between *E*. *coli* strains [15]. However, ST toxin can also be encoded by transposons, which are mobile genetic elements as well [1]. ETEC plasmids can carry both ETEC colonization factors and *est* [12,51]. Some ETEC strains are negative for colonization factors [24], as was the case with our STEC/ETEC strains. The survey of plasmid-associated sequences indicated that *estIa* in the STEC/ETEC hybrids may be associated with plasmids. Nevertheless, it is not surprising that both ETEC and STEC strains can arise in different phylogenetic groups and that they do not necessarily have a clonal lineage. Some commensal strains may have pathogenic potential since certain parts of their genomes may act as genetic repositories for virulence factors [10]. Acquisition of appropriate pathogenic features may cause a transformation of a commensal strain to a pathogen or a strain of one pathogroup to a hybrid pathogroup.

## Conclusions

The comparative genomics of the STEC/ETEC hybrid strains showed that STEC- and ETEC-associated virulence genes can co-exists in strains originating from different phylogenetic lineages. Whole genome sequencing techniques enable fast typing and possibility to screen several genetic markers simultaneously making it easy to detect virulence genes associated with several pathogroups. An infection with a hybrid pathogenic strain may result in more severe disease in a patient. These strains may also have increased spreading potential. Therefore, their emergence should be taken into account in modern strain typing and in epidemiological surveillance of *E*. *coli* infections.

## Supporting Information

S1 Fig Whole genome restriction map similarities. (A) Comparison between IH53473 and IH57218, (B) comparison between IH53473 and FE95160, and (C) comparison between IH57218 and FE95160.
(TIF)

S1 Table The STEC/ETEC genomes sequenced and the reference genomes used in this study. (DOC)

S2 Table Extracted virulence genes. (DOC)

## References

[1] Nataro JP, Kaper JB. Diarrheagenic *Escherichia coli* . Clin Microbiol Rev. 1998;11: 142–201. 9457432 10.1128/cmr.11.1.142 PMC121379

[2] Müller D, Greune L, Heusipp G, Karch H, Fruth A, Tschäpe H, et al Identification of unconventional intestinal pathogenic *Escherichia coli* isolates expressing intermediate virulence factor profiles by using a novel single-step multiplex PCR. Appl Environ Microbiol. 2007;73: 3380–3390. 17400780 10.1128/AEM.02855-06 PMC1907121

[3] Mellmann A, Harmsen D, Cummings CA, Zentz EB, Leopold SR, Rico A, et al Prospective genomic characterization of the German enterohemorrhagic *Escherichia coli* O104:H4 outbreak by rapid next generation sequencing technology. PLoS One 2011;6: e22751 10.1371/journal.pone.0022751 21799941 PMC3140518

[4] Karch H, Denamur E, Dobrindt U, Finlay BB, Hengge R, Johannes L, et al The enemy within us: lessons from the 2011 European *Escherichia coli* O104:H4 outbreak. EMBO Mol Med. 2012;4: 841–848. 10.1002/emmm.201201662 22927122 PMC3491817

[5] Vu-Khac H, Holoda E, Pilipcinec E, Blanco M, Blanco JE, Dahbi G, et al Serotypes, virulence genes, intimin types and PFGE profiles of *Escherichia coli* isolated from piglets with diarrhoea in Slovakia. Vet J. 2007;174: 176–187. 16956777 10.1016/j.tvjl.2006.05.019

[6] Fratamico PM, Bhagwat AA, Injaian L, Fedorka-Cray PJ. Characterization of shiga toxin-producing *Escherichia coli* strains isolated from swine feces. Foodborne Pathog Dis. 2008;5: 827–838. 10.1089/fpd.2008.0147 18991545

[7] Prager R, Fruth A, Busch U, Tietze E. Comparative analysis of virulence genes, genetic diversity, and phylogeny of Shiga toxin 2g and heat-stable enterotoxin STIa encoding *Escherichia coli* isolates from humans, animals, and environmental sources. Int J Med Microbiol. 2011;301: 181–191. 10.1016/j.ijmm.2010.06.003 20728406

[8] Nyholm O, Heinikainen S, Pelkonen S, Hallanvuo S, Haukka K, Siitonen A. Hybrids of Shigatoxigenic and enterotoxigenic *Escherichia coli* (STEC/ETEC) among human and animal isolates in Finland. Zoonoses Public Health 2015 10.1111/zph.12177 25571907

[9] Martikainen O, Kagambèga A, Bonkoungou IJ, Barro N, Siitonen A, Haukka K. Characterization of shigatoxigenic *Escherichia coli* strains from Burkina Faso. Foodborne Pathog Dis. 2012;9: 1015–1021. 10.1089/fpd.2012.1228 23134285

[10] Rasko DA, Rosovitz MJ, Myers GS, Mongodin EF, Fricke WF, Gajer P, et al The pangenome structure of *Escherichia coli*: comparative genomic analysis of *E*. *coli* commensal and pathogenic isolates. J Bacteriol. 2008;190: 6881–6893. 10.1128/JB.00619-08 18676672 PMC2566221

[11] Ogura Y, Ooka T, Iguchi A, Toh H, Asadulghani M, Oshima K, et al Comparative genomics reveal the mechanism of the parallel evolution of O157 and non-O157 enterohemorrhagic *Escherichia coli* . Proc Natl Acad Sci U S A. 2009;106: 17939–17944. 10.1073/pnas.0903585106 19815525 PMC2764950

[12] Sahl JW, Steinsland H, Redman JC, Angiuoli SV, Nataro JP, Sommerfelt H, et al A comparative genomic analysis of diverse clonal types of enterotoxigenic *Escherichia coli* reveals pathovar-specific conservation. Infect Immun. 2011;79: 950–960. 10.1128/IAI.00932-10 21078854 PMC3028850

[13] Cooper KK, Mandrell RE, Louie JW, Korlach J, Clark TA, Parker CT, et al Comparative genomics of enterohemorrhagic *Escherichia coli* O145:H28 demonstrates a common evolutionary lineage with *Escherichia coli* O157:H7. BMC Genomics 2014;15: 17 10.1186/1471-2164-15-17 24410921 PMC3893438

[14] Steyert SR, Sahl JW, Fraser CM, Teel LD, Scheutz F, Rasko DA. Comparative genomics and stx phage characterization of LEE-negative Shiga toxin-producing *Escherichia coli* . Front Cell Infect Microbiol. 2012;2: 133 10.3389/fcimb.2012.00133 23162798 PMC3491183

[15] Turner SM, Chaudhuri RR, Jiang ZD, DuPont H, Gyles C, Penn CW, et al Phylogenetic comparisons reveal multiple acquisitions of the toxin genes by enterotoxigenic *Escherichia coli* strains of different evolutionary lineages. J Clin Microbiol. 2006;44: 4528–4536. 17050815 10.1128/JCM.01474-06 PMC1698409

[16] Chen Q, Savarino SJ, Venkatesan MM. Subtractive hybridization and optical mapping of the enterotoxigenic *Escherichia coli* H10407 chromosome: isolation of unique sequences and demonstration of significant similarity to the chromosome of *E*. *coli* K-12. Microbiology 2006;152: 1041–1054. 16549668 10.1099/mic.0.28648-0

[17] von Mentzer A, Connor TR, Wieler LH, Semmler T, Iguchi A, Thomson N5, et al Identification of enterotoxigenic *Escherichia coli* (ETEC) clades with long-term global distribution. Nat Genet. 2014;46: 1321–1326. 10.1038/ng.3145 25383970

[18] Rasko DA, Webster DR, Sahl JW, Bashir A, Boisen N, Scheutz F, et al Origins of the *E*. *coli* strain causing an outbreak of hemolytic-uremic syndrome in Germany. N Engl J Med. 2011;365: 709–717. 10.1056/NEJMoa1106920 21793740 PMC3168948

[19] Cuccuru G, Orsini M, Pinna A, Sbardellati A, Soranzo N, Travaglione A, et al Orione, a web-based framework for NGS analysis in microbiology. Bioinformatics. 2014;30: 1928–1929. 10.1093/bioinformatics/btu135 24618473 PMC4071203

[20] Seemann T. Prokka: rapid prokaryotic genome annotation. Bioinformatics. 2014;30: 2068–2069. 10.1093/bioinformatics/btu153 24642063

[21] Schwan WR, Briska A, Stahl B, Wagner TK, Zentz E, Henkhaus J, et al Use of optical mapping to sort uropathogenic *Escherichia coli* strains into distinct subgroups. Microbiology 2010;156: 2124–2135. 10.1099/mic.0.033977-0 20378655 PMC3068680

[22] Rodas C, Iniguez V, Qadri F, Wiklund G, Svennerholm AM, Sjöling A. Development of multiplex PCR assays for detection of enterotoxigenic *Escherichia coli* colonization factors and toxins. J Clin Microbiol. 2009;47: 1218–1220. 10.1128/JCM.00316-09 19244463 PMC2668350

[23] Vidal RM, Valenzuela P, Baker K, Lagos R, Esparza M, Livio S, et al Characterization of the most prevalent colonization factor antigens present in Chilean clinical enterotoxigenic *Escherichia coli* strains using a new multiplex polymerase chain reaction. Diagn Microbiol Infect Dis. 2009;65: 217–223. 10.1016/j.diagmicrobio.2009.07.005 19733027

[24] Del Canto F, Valenzuela P, Cantero L, Bronstein J, Blanco JE, Blanco J, et al Distribution of classical and nonclassical virulence genes in enterotoxigenic *Escherichia coli* isolates from Chilean children and tRNA gene screening for putative insertion sites for genomic islands. J Clin Microbiol. 2011;49: 3198–3203. 10.1128/JCM.02473-10 21775541 PMC3165568

[25] Statens Serum Institute. Identification of three *vtx1* and seven *vtx2* subtypes of Verocytotoxin encoding genes of *Escherichia coli* by conventional PCR amplification, Version 6. Available: http://www.ssi.dk/English/HealthdataandICT/National%20Reference%20Laboratories/Bacteria/~/media/Indhold/EN%20-%20engelsk/Public%20Health/National%20Reference%20Laboratories/vtx%20detection%20%20subtyping%20protocolrev6final.ashx. Accessed 14 January 2015.

[26] Wirth T, Falush D, Lan R, Colles F, Mensa P, Wieler LH, et al Sex and virulence in *Escherichia coli*: an evolutionary perspective. Mol Microbiol. 2006;60: 1136–1151. 16689791 10.1111/j.1365-2958.2006.05172.x PMC1557465

[27] Clermont O, Christenson JK, Denamur E, Gordon DM. The Clermont *Escherichia coli* phylo-typing method revisited: improvement of specificity and detection of new phylo-groups. Environ Microbiol Rep. 2013;5: 58–65. 10.1111/1758-2229.12019 23757131

[28] Clermont O, Gordon DM, Brisse S, Walk ST, Denamur E. Characterization of the cryptic *Escherichia* lineages: rapid identification and prevalence. Environ Microbiol. 2011;13: 2468–2477. 10.1111/j.1462-2920.2011.02519.x 21651689

[29] Ballmer K, Korczak BM, Kuhnert P, Slickers P, Ehricht R, Hächler H. Fast DNA serotyping of *Escherichia coli* by use of an oligonucleotide microarray. J Clin Microbiol. 2007;45: 370–379. 17108073 10.1128/JCM.01361-06 PMC1829071

[30] Geue L, Monecke S, Engelmann I, Braun S, Slickers P, Ehricht R. Rapid microarray-based DNA genoserotyping of *Escherichia coli* . Microbiol Immunol. 2014;58: 77–86. 10.1111/1348-0421.12120 24298918

[31] Zhou Y, Liang Y, Lynch KH, Dennis JJ, Wishart DS. PHAST: a fast phage search tool. Nucleic Acids Res. 2011;39(Web Server issue): W347–W352. 10.1093/nar/gkr485 21672955 PMC3125810

[32] Zhang Z, Schwartz S, Wagner L, Miller W. A greedy algorithm for aligning DNA sequences. J Comput Biol. 2000;7: 203–214. 10890397 10.1089/10665270050081478

[33] Sjöling Å, Wiklund G, Savarino SJ, Cohen DI, Svennerholm AM. Comparative analyses of phenotypic and genotypic methods for detection of enterotoxigenic *Escherichia coli* toxins and colonization factors. J Clin Microbiol. 2007;45: 3295–3301. 17687011 10.1128/JCM.00471-07 PMC2045327

[34] Svennerholm AM, Wikström M, Lindblad M, Holmgren J. Monoclonal antibodies against *Escherichia coli* heat-stable toxin (STa) and their use in a diagnostic ST ganglioside GM1-enzyme-linked immunosorbent assay. J Clin Microbiol. 1986;24: 585–590. 2429984 10.1128/jcm.24.4.585-590.1986 PMC268976

[35] Carattoli A, Zankari E, García-Fernández A, Voldby Larsen M, Lund O, Villa L, et al *In silico* detection and typing of plasmids using PlasmidFinder and plasmid multilocus sequence typing. Antimicrob Agents Chemother. 2014;58: 3895–3903. 10.1128/AAC.02412-14 24777092 PMC4068535

[36] Katoh H, Standley DM. MAFFT Multiple Sequence Alignment Software Version 7: Improvements in Performance and Usability. Mol Biol Evol. 2013;30: 772–780. 10.1093/molbev/mst010 23329690 PMC3603318

[37] Shepard SM, Danzeisen JL, Isaacson RE, Seemann T, Achtman M, Johnson TJ. Genome sequences and phylogenetic analysis of K88- and F18-positive porcine enterotoxigenic *Escherichia coli* . J Bacteriol. 2012;194: 395–405. 10.1128/JB.06225-11 22081385 PMC3256668

[38] Schubert S, Rakin A, Karch H, Carniel E, Heesemann J. Prevalence of the "high-pathogenicity island" of *Yersinia* species among *Escherichia coli* strains that are pathogenic to humans. Infect Immun. 1998;66: 480–485. 9453599 10.1128/iai.66.2.480-485.1998 PMC107931

[39] Boisen N, Ruiz-Perez F, Scheutz F, Krogfelt KA, Nataro JP. Short report: high prevalence of serine protease autotransporter cytotoxins among strains of enteroaggregative *Escherichia coli* . Am J Trop Med Hyg. 2009;80: 294–301. 19190229 PMC2660206

[40] Paiva de Sousa C, Dubreuil JD. Distribution and expression of the *astA* gene (EAST1 toxin) in *Escherichia coli* and *Salmonella* . Int J Med Microbiol. 2001;291: 15–20. 11403406 10.1078/1438-4221-00097

[41] Toval F, Köhler CD, Vogel U, Wagenlehner F, Mellmann A, Fruth A, et al Characterization of *Escherichia coli* isolates from hospital inpatients or outpatients with urinary tract infection. J Clin Microbiol. 2014;52: 407–418. 10.1128/JCM.02069-13 24478469 PMC3911323

[42] Wells TJ, Totsika M, Schembri MA. Autotransporters of *Escherichia coli*: a sequence-based characterization. Microbiology. 2010;156: 2459–2469. 10.1099/mic.0.039024-0 20447993

[43] Olesen B, Hansen DS, Nilsson F, Frimodt-Møller J, Leihof RF, Struve C, et al Prevalence and characteristics of the epidemic multiresistant *Escherichia coli* ST131 clonal group among extended-spectrum beta-lactamase-producing *E*. *coli* isolates in Copenhagen, Denmark. J Clin Microbiol. 2013;51: 1779–1785. 10.1128/JCM.00346-13 23554186 PMC3716056

[44] Telli M, Guiral E, Martínez JA, Almela M, Bosch J, Vila J, et al Prevalence of enterotoxins among *Escherichia coli* isolates causing bacteraemia. FEMS Microbiol Lett. 2010;306: 117–121. 10.1111/j.1574-6968.2010.01945.x 20529132

[45] Lloyd AL, Smith SN, Eaton KA, Mobley HL. Uropathogenic *Escherichia coli* suppresses the host inflammatory response via pathogenicity island genes *sisA* and *sisB* . Infect Immun. 2009;77: 5322–5333. 10.1128/IAI.00779-09 19797063 PMC2786477

[46] Toval F, Schiller R, Meisen I, Putze J, Kouzel IU, Zhang W, et al Characterization of urinary tract infection-associated Shiga toxin-producing *Escherichia coli* . Infect Immun. 2014;82: 4631–4642. 10.1128/IAI.01701-14 25156739 PMC4249321

[47] Shen J, Rump L, Ju W, Shao J, Zhao S, Brown E, et al Virulence characterization of non-O157 Shiga toxin-producing *Escherichia coli* isolates from food, humans and animals. Food Microbiol. 2015;50: 20–27. 10.1016/j.fm.2015.02.007 25998811

[48] Ratiner Y. Serotyping of *Escherichia coli* antigens In: Stain G, Fünsfstück R, editors. *Harnwegsinfektion*, *Aktuelle Gesichtspunkte zur Pathogenese*, *Diagnostic and Therapie*. II Wissenschliches Symposium, pmi-Verlag GmbH, Frankfurt am Main, Germany; 1991 pp. 47–51.

[49] Serra-Moreno R, Jofre J, Muniesa M. Insertion site occupancy by *stx* _*2*_ bacteriophages depends on the locus availability of the host strain chromosome. J Bacteriol. 2007;189: 6645–6654. 17644594 10.1128/JB.00466-07 PMC2045183

[50] Schmidt H. Shiga-toxin-converting bacteriophages. Res Microbiol. 2001;152: 687–695. 11686382 10.1016/s0923-2508(01)01249-9

[51] Wolf MK. Occurrence, distribution, and associations of O and H serogroups, colonization factor antigens, and toxins of enterotoxigenic *Escherichia coli* . Clin Microbiol Rev. 1997;10: 569–584. 9336662 10.1128/cmr.10.4.569 PMC172934

