# Supplementary figures and images for: Comparative Genomics and Characterization of Hybrid Shigatoxigenic and Enterotoxigenic Escherichia coli (STEC/ETEC) Strains
Source: PLoS One. 2015 Aug 27;10(8):e0135936. doi: 10.1371/journal.pone.0135936 (PMC4551483; doi:10.1371/journal.pone.0135936)

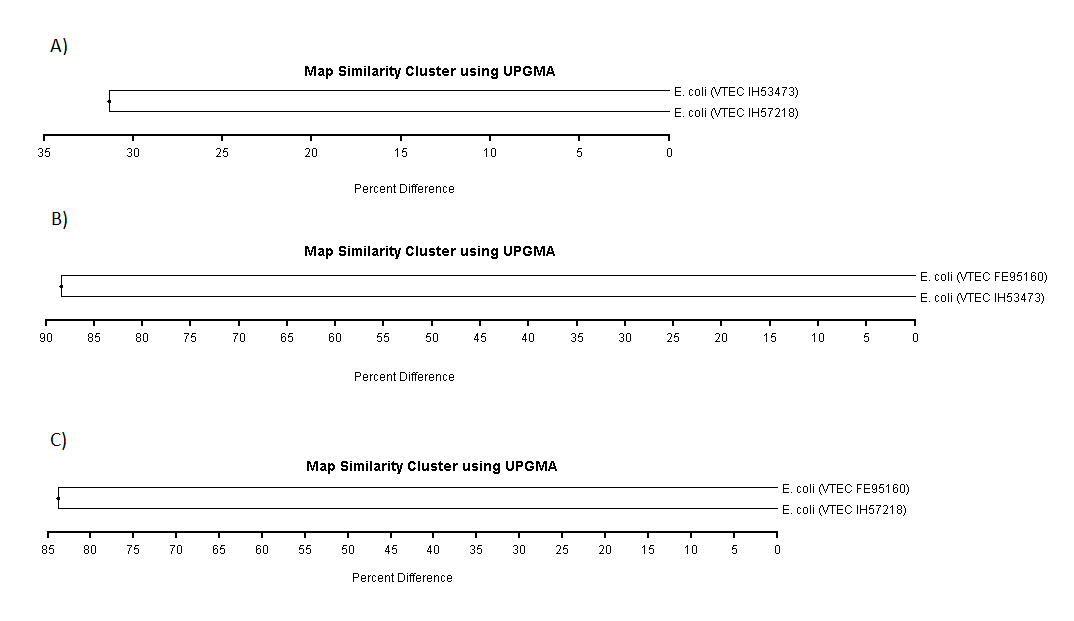

Supplement: S1 Fig — (A) Comparison between IH53473 and IH57218, (B) comparison between IH53473 and FE95160, and (C) comparison between IH57218 and FE95160. (TIF) [file pone.0135936.s001.tif]
