# Supplementary material for: Comparative Genomics and Characterization of Hybrid Shigatoxigenic and Enterotoxigenic Escherichia coli (STEC/ETEC) Strains
Source: PLoS One. 2015 Aug 27;10(8):e0135936. doi: 10.1371/journal.pone.0135936 (PMC4551483; doi:10.1371/journal.pone.0135936)
Supplement: S1 Table — (DOC) [file pone.0135936.s002.doc]

**S1 Table. The STEC/ETEC genomes sequenced and the reference genomes used in this study.**

| **Genome** | **Accession** | **Assembly status** |
| --- | --- | --- |
| STEC/ETEC O101:H- IH53473 | LFZH00000000 | Draft |
| STEC/ETEC O2:H27 IH57218 | LFZJ00000000 | Draft |
| STEC/ETEC O2:H2 FE95160 | LFZI00000000 | Draft |
| STEC O157:H7 Sakai | NC_002695.1 | Complete |
| STEC O26:H11 11368 | NC_013361.1 | Complete |
| STEC O5:H- 97.0246 | AEZJ00000000.2 | Draft |
| STEC O11:H- 97.0259 | AEZL00000000.2 | Draft |
| STEC O76:H- 1.2264 | AEZO00000000.2 | Draft |
| STEC O145:H- 08-4270 | JHKV00000000.1 | Draft |
| STEC O174:H8 04-3038 | JHOC00000000.1 | Draft |
| STEC O2:H25 STEC_7v | AEXD00000000.1 | Draft |
| STEC O73:H18 C165-02 | AFDR00000000.1 | Draft |
| STEC O139:H1 S1191 | AFEA00000000.1 | Draft |
| STEC O8:H19 MHI813 | AFDZ00000000.1 | Draft |
| STEC O111:H- 11128 | NC_013364.1 | Complete |
| STEC O2:H6 09-05501 | ERR355896 | Draft |
| STEC O103:H2 12009 | NC_013353.1 | Complete |
| STEC O2:H4 1.2741 | AEZI00000000.2 | Draft |
| ETEC O78:H11 H10407 | FN649414.1 | Complete |
| ETEC 179100 | APZJ00000000.1 | Draft |
| ETEC O101:H- B41 | AFAH00000000.2 | Draft |
| ETEC O139:H28 E24377A | NC_009801.1 | Complete |
| ETEC O6:H16 TW10598 | AELA00000000.1 | Draft |
| ETEC O?:H5 TW10722 | AELB00000000.1 | Draft |
| ETEC O19:H45 TW11681 | AELD00000000 | Draft |
| ETEC O114:H49 TW10828 | AELC00000000 | Draft |
| ETEC O78:H9 TW14425 | AELE00000000 | Draft |
| ETEC O149 UMNK88 | NC_017641.1 | Complete |
| ETEC O147 UMNF18 | AGTD01000001.1 | Draft |
| ETEC O6 E8 | ERS038927 | Draft |
| ETEC O6 E66 | ERS044460 | Draft |
| ETEC O78 E36 | ERS044458 | Draft |
| ETEC O25 E135 | ERS044471 | Draft |
| ETEC O115 E21 | ERS044456 | Draft |
| ETEC ON3 E562 | ERS038940 | Draft |
| ETEC O169 E344 | ERS038934 | Draft |
| ETEC O148 E222 | ERS077576 | Draft |
| ETEC O27 E220 | ERS044475 | Draft |
| ETEC O114 E934 | ERS077651 | Draft |
| ETEC O159 E159 | ERS077713 | Draft |
| ETEC O15 E330 | ERS077581 | Draft |
| ETEC O112ab E399 | ERS077593 | Draft |
| ETEC ON5 E620 | ERS077610 | Draft |
| ETEC O148:H28 B7A | AAJT00000000 | Draft |
| EPEC O111:H9 E110019 | AAJW00000000.2 | Draft |
| EPEC O127:H6 E2348/69 | NC_011601.1 | Complete |
| EPEC O55:H7 CB9615 | NC_013941.1 | Complete |
| Shigella sonnei Ss046 | NC_007384.1 | Complete |
| Shigella flexneri 2a str. 301 | NC_004337.2 | Complete |
| Shigella boydii Sb227 | NC_007613.1 | Complete |
| Shigella dysenteriae Sd197 | NC_007606.1 | Complete |
| EAEC O44:H18 042 | NC_017626.1 | Complete |
| EAEC ONT:H33 C48/93 | AHAX00000000.1 | Draft |
| EAEC O104:H4 55989 | NC_011748.1 | Complete |
| EAEC/STEC O104:H4 2011C-3493 | NC_018658.1 | Complete |
| EAEC/STEC O104:H4 2009EL-2071 | NC_018661.1 | Complete |
| Lab adapted B str. REL606 | CP000819.1 | Complete |
| Lab adapted K-12 substr. MG1655 | NC_000913.3 | Complete |
| Lab adapted W | NC_017635.1 | Complete |
| Commensal SE11 | NC_011415.1 | Complete |
| Commensal ED1a | NC_011745.1 | Complete |
| Commensal IAI1 | NC_011741.1 | Complete |
| Commensal HS | NC_009800.1 | Complete |
| Commensal SE15 | NC_013654.1 | Complete |
| Commensal P12b | NC_017663.1 | Complete |
| Environmental SMS-3-5 | NC_010498.1 | Complete |
| UPEC 536 | NC_008253.1 | Complete |
| UPEC IAI39 | NC_011750.1 | Complete |
| ExPEC JJ1886 | NC_022648.1 | Complete |
| ExPEC ABU 83972 | NC_017631.1 | Complete |
| MNEC IHE3034 | NC_017628.1 | Complete |
| MNEC CE10 | NC_017646.1 | Complete |
| AIEC LF82 | NC_011993.1 | Complete |
| AIEC UM146 | NC_017632.1 | Complete |
| APEC O1 | NC_008563.1 | Complete |
| APEC O78 | NC_020163.1 | Complete |
