# Supplementary material for: Comparative Genomics and Characterization of Hybrid Shigatoxigenic and Enterotoxigenic Escherichia coli (STEC/ETEC) Strains
Source: PLoS One. 2015 Aug 27;10(8):e0135936. doi: 10.1371/journal.pone.0135936 (PMC4551483; doi:10.1371/journal.pone.0135936)
Supplement: S2 Table — (DOC) [file pone.0135936.s003.doc]

**S2** Table. Extracted virulence genes.

| **Gene/Target** | **Product/Function** | **Reference** | **Accession** | **Locus_tag in the reference** |
| --- | --- | --- | --- | --- |
| stx1A | Shiga toxin 1 subunit A | Escherichia coli O157:H7 str. Sakai chromosome | NC_002695 | ECs2974 |
| stx1B | Shiga toxin 1 subunit b | Escherichia coli O157:H7 str. Sakai chromosome | NC_002695 | ECs2973 |
| stx2A | Shiga toxin 2 subunit A | Escherichia coli O157:H7 str. Sakai chromosome | NC_002695 | ECs1205 |
| stx2B | Shiga toxin 2 subunit b | Escherichia coli O157:H7 str. Sakai chromosome | NC_002695 | ECs1206 |
| eltA (elt-II) | Heat-labile enterotoxin A chain precursor (lt-a porcine, ltp-a) | Escherichia coli ETEC H10407 plasmid p666 | NC_017722 | ETEC_p666_p0660 |
| eltB (elt-I) | Heat-labile enterotoxin B chain precursor (lt-b human, lth-b) | Escherichia coli ETEC H10407 plasmid p666 | NC_017722 | ETEC_p666_p0650 |
| sta1 (estIa) | Heat-stable enterotoxin sti-a/st-p precursor | Escherichia coli ETEC H10407 plasmid p666 | NC_017722 | ETEC_p666_p0750 |
| sta2 (estIb) | Heat-stable enterotoxin A2 precursor | Escherichia coli ETEC H10407 plasmid p948 | NC_017724 | ETEC_p948_0360 |
| astA | EAEC heat-stable enterotoxin I | Escherichia coli enteroaggregative heat-stable enterotoxin 1 (astA) gene, complete cds | L11241 | - |
| hlyA (ehxA) | Hemolysin A | Escherichia coli O157:H7 str. Sakai plasmid pO157 | NC_002128 | pO157p18 |
| hlyB (ehxB) | Hemolysin B | Escherichia coli O157:H7 str. Sakai plasmid pO157 | NC_002128 | pO157p19 |
| hlyC (ehxC) | Hemolysin C | Escherichia coli O157:H7 str. Sakai plasmid pO157 | NC_002128 | pO157p17 |
| hlyD (ehxD) | Hemolysin D | Escherichia coli O157:H7 str. Sakai plasmid pO157 | NC_002128 | pO157p20 |
| eae | Intimin | Escherichia coli O26:H11 str. 11368 chromosome | NC_013361 | ECO26_5280 |
| escV | T3SS structure protein EscV | Escherichia coli O26:H11 str. 11368 chromosome | NC_013361 | ECO26_5270 |
| tir | Translocated intimin reseptor Tir | Escherichia coli O26:H11 str. 11368 chromosome | NC_013361 | ECO26_5278 |
| espA | Translocator EspA | Escherichia coli O26:H11 str. 11368 chromosome | NC_013361 | ECO26_5283 |
| espB | Translocator EspB | Escherichia coli O26:H11 str. 11368 chromosome | NC_013361 | ECO26_5285 |
| espD | Translocator EspD | Escherichia coli O26:H11 str. 11368 chromosome | NC_013361 | ECO26_5284 |
| espF | T3SS effector EspF | Escherichia coli O26:H11 str. 11368 chromosome | NC_013361 | ECO26_5289 |
| espG | T3SS effector EspG | Escherichia coli O26:H11 str. 11368 chromosome | NC_013361 | ECO26_5250 |
| espH | T3SS effector EspH | Escherichia coli O26:H11 str. 11368 chromosome | NC_013361 | ECO26_5275 |
| espZ | T3SS effector EspZ | Escherichia coli O26:H11 str. 11368 chromosome | NC_013361 | ECO26_5268 |
| espP | Extracellular serine protease, autotransporter, SPATE | Escherichia coli EH41 plasmid pO113 | NC_007365 | LH0167 |
| sepA | Serine protease SepA precursor, EatA homologue, SPATE | Escherichia coli 55989 plasmid 55989p | NC_011752 | pEC55989_0062 |
| sat | Serine protease Sat precursot, SPATE | Escherichia coli 55989 plasmid 55989p | NC_011752 | pEC55989_0068 |
| epeA | Autotransporter protease, SPATE | Escherichia coli EH41 plasmid pO113 | NC_007365 | LH0053 |
| pet | Serine protease, SPATE | Escherichia coli 042 plasmid pAA | FN554767 | EC042_pAA035 |
| pic | Serine protease, autotransporter, SPATE | Escherichia coli 042 chromosome | FN554766 | EC042_4593 |
| eatA | Serine protease EatA, SPATE | Escherichia coli ETEC H10407 plasmid p948 | NC_017724 | ETEC_p948_0020 |
| sigA | Serine protease, SPATE | Shigella flexneri 2a str. 301 chromosome | NC_004337 | SF2968 |
| tsh | Serine protease, SPATE | Escherichia coli plasmid pAPEC-1 IS100 putative transposases, IS30 putative transposase, IS911 putative transposase, Tsh (tsh) and unknown genes, complete cds | AF218073 | - |
| vat | Serine protease, SPATE | Escherichia coli strain Ec222 tRNA-Trp gene and pathogenicity island, complete sequence; YagU gene, complete cds; YkgJ gene, partial cds; and unknown genes | AY151282 | - |
| espC | Serine protease, SPATE | Escherichia coli enterotoxin EspC (espC) gene, complete cds | AF297061 | - |
| O3M_04615 | Subtilisin-like serine protease | Escherichia coli O104:H4 str. 2009EL-2050 chromosome | NC_018650 | O3M_04615 |
| etpA | Two-partner secreted adhesin EtpA | Escherichia coli ETEC H10407 plasmid p948 | NC_017724 | ETEC_p948_0110 |
| saa | STEC autoagglutinating adhesin | Escherichia coli EH41 plasmid pO113 | NC_007365 | LH0174 |
| iha | IrgA-homologue adhesin | Escherichia coli EH41 plasmid pO113 | NC_007365 | LH0178 |
| tibA | Adhesin/invasin tibA precursor; Glycoprotein TibA, which has been implicated in bacterial autoaggregation and biofilm formation | Escherichia coli ETEC H10407 chromosome | FN649414 | ETEC_2141 |
| eaeH | Putative adhesin | Escherichia coli strain H10407 EaeH gene, complete cds | DQ109813 | - |
| yfaL | Putative outer membrane autotransporter adhesin | Escherichia coli E24377A chromosome | CP000800 | EcE24377A_2528 |
| sfaA | S-fimbrial adhesin, major subunit SfaA | Escherichia coli IHE3034 chromosome | CP001969 | ECOK1_1098 |
| aidaA | Autotransporter adhesin, diffuse adherence adhesin | Escherichia coli strain 2787 plasmid AIDA-associated heptosyltransferase (aah) and diffuse adherence adhesin (aidA) genes, complete cds | GU810159 | - |
| aah | Autotransporter adhesin, diffuse adherence adhesin | Escherichia coli strain 2787 plasmid AIDA-associated heptosyltransferase (aah) and diffuse adherence adhesin (aidA) genes, complete cds | GU810159 | - |
| subA | Subtilase cytotoxin subunit A | Escherichia coli EH41 plasmid pO113 | NC_007365 | LH0183 |
| subB | Subtilase cytotoxin subunit B | Escherichia coli EH41 plasmid pO113 | NC_007365 | LH0182 |
| toxB | Toxin B | Escherichia coli O157:H7 str. Sakai plasmid pO157 | NC_002128 | pO157p58 |
|  |  |  |  |  |
| ccdB | Post-segregation toxin (cytotoxic protein) | Escherichia coli 042 plasmid pAA | FN554767 | EC042_pAA017 |
| clyA | Cytolysin A | Escherichia coli ClyA (clyA) gene, complete cds | AF240780.1 | - |
| tia | Tia invasion determinant | Escherichia coli O7:K1 str. CE10 chromosome | NC_017646 | CE10_4869 |
| leoA | Virulence associated protein, GTPase, required for maximal secretion of the heat-labile enterotoxin | Escherichia coli ETEC H10407 chromosome | FN649414 | ETEC_3904 |
| aggR | Transcriptional activator | Escherichia coli 042 plasmid pAA | FN554767 | EC042_pAA052 |
| aap | Dispersin | Escherichia coli 042 plasmid pAA | FN554767 | EC042_pAA055 |
| aafA | Major fimbrial subunit of aggregative adherence fimbria II | Escherichia coli 042 plasmid pAA | FN554767 | EC042_pAA048 |
| aafB | Afimbrial adhesin | Escherichia coli 042 plasmid pAA | FN554767 | EC042_pAA030 |
| aafC | Aggregative adherence fimbria II usher protein | Escherichia coli 042 plasmid pAA | FN554767 | EC042_pAA031 |
| aafD | Chaperone protein | Escherichia coli 042 plasmid pAA | FN554767 | EC042_pAA046 |
| aatA | Permease | Escherichia coli 042 plasmid pAA | FN554767 | EC042_pAA007 |
| aatP | Outer membrane protein | Escherichia coli 042 plasmid pAA | FN554767 | EC042_pAA008 |
| sfpA | SfpA; major fimbrial protein precursor | Escherichia coli plasmid pSFO157 | NC_009602 | SF0157_p06 |
| aggA | Fimbrial subunit | Escherichia coli aggregative adherence fimbria I gene cluster | U12894 | - |
| aggB | Fimbrial subunit | Escherichia coli aggregative adherence fimbria I gene cluster | U12894 | - |
| aggC | Fimbrial subunit | Escherichia coli aggregative adherence fimbria I gene cluster | U12894 | - |
| aggD | Fimbrial subunit | Escherichia coli aggregative adherence fimbria I gene cluster | U12894 | - |
| aatA | Outer membrane protein | Escherichia coli 042 plasmid pAA | FN554767 | EC042_pAA008 |
| aatB | Conserved hypothetical protein | Escherichia coli 042 plasmid pAA | FN554767 | EC042_pAA009 |
| aatC | ABC transporter, ATP-binding protein | Escherichia coli 042 plasmid pAA | FN554767 | EC042_pAA010 |
| aatD | Putative membrane protein | Escherichia coli 042 plasmid pAA | FN554767 | EC042_pAA011 |
| agg3A | Adhesin protein | Escherichia coli str. 55989 plasmid 55989p | CU928159.2 | pEC55989_0084 |
| agg3B | Putative invasin | Escherichia coli str. 55989 plasmid 55989p | CU928159.2 | pEC55989_0085 |
| agg3C | Outer membrane usher protein | Escherichia coli str. 55989 plasmid 55989p | CU928159.2 | pEC55989_0086 |
| agg3D | Chaperone protein | Escherichia coli str. 55989 plasmid 55989p | CU928159.2 | pEC55989_0087 |
| aai pathogenicity island | Type VI secretion system | Escherichia coli 042 chromosome | FN554766 | EC042_4562 to EC042_4577 |
| ShET-2 | Shigella enterotoxin ShET-2 domain containing protein | Escherichia coli O104:H4 str. 2009EL-2050 chromosome | NC_018650 | O3M_23715 |
| O3K_25787 | Beta-lactamase | Escherichia coli O104:H4 str. 2011C-3493 plasmid pESBL-EA11 | NC_018659 | O3K_25787 |
| O3K_25812 | Beta-lactamase TEM | Escherichia coli O104:H4 str. 2011C-3493 plasmid pESBL-EA11 | NC_018659 | O3K_25812 |
| ter | Tellurite resistance | Escherichia coli O157:H7 str. Sakai chromosome | NC_002695 | ECs2035 |
|  |  |  |  |  |
| ecpA | Common pilus subunit | Escherichia coli strain B2C EcpA (ecpA) gene, complete cds | FJ210912.1 | - |
| katP | Catalase-peroxidase KatP | Escherichia coli O26:H11 str. 11368 plasmid pO26_1 DNA | AP010954 | ECO26_p1-46 |
| capU | Hexosyltransferase homolog, similar to Shigella flexneri VirK | Escherichia coli plasmid pAA2 Shf (shf), hexosyltransferase homolog (capU), and VirK (virK) genes, complete cds | AF134403 | - |
| fimH | Type 1 fimbria | Escherichia coli E24377A chromosome | CP000800 | EcE24377A_4917 |
| shiA | Shikimate transporter | Escherichia coli CFT073 chromosome | AE014075 | C2443 |
| irp1 | Yersiniabactin biosynthetic protein | Yersinia pestis CO92 chromosome | NC_003143 | YPO1910 |
| irp2 | Yersiniabactin biosynthetic protein | Yersinia pestis CO92 chromosome | NC_003143 | YPO1911 |
| fyuA | Pesticin, yersiniabactin receptor protein | Yersinia pestis CO92 chromosome | NC_003143 | YPO1906 |
| lngA | Longus pilus structural subunit | Escherichia coli strain M526-C6B longus pilus structural subunit (lngA) gene, complete cds | EU107107 | - |
